# Topical Innovation: A Report on Epinastine Eyelid Cream for Patients With Mild Atopic Keratoconjunctivitis

**DOI:** 10.7759/cureus.86792

**Published:** 2025-06-26

**Authors:** Tatsuya Mimura, Kazumi Fukagawa

**Affiliations:** 1 Ophthalmology, Teikyo University School of Medicine, Tokyo, JPN; 2 Ophthalmology, Tsurumi University Dental Hospital, Yokohama, JPN; 3 Ophthalmology, Ryogoku Eye Clinic, Tokyo, JPN; 4 Ophthalmology, Keio University School of Medicine, Tokyo, JPN

**Keywords:** allergic conjunctivitis, atopic dermatitis, atopic keratoconjunctivitis, epinastine, eyelid cream

## Abstract

Allergic conjunctivitis is a common ocular disorder that significantly impacts patients' quality of life. Conventional treatments, primarily anti-allergic and corticosteroid eye drops, require frequent administration, posing adherence challenges. Poor compliance is a major barrier to effective management, particularly in pediatric and elderly patients. Recently, a novel 0.5% epinastine topical eyelid cream, designed for once-daily application, has been introduced as an alternative treatment. However, no clinical reports for atopic keratoconjunctivitis have assessed its efficacy and safety in real-world practice. This study presents a clinical report on the use of 0.5% epinastine topical eyelid cream in three patients with allergic conjunctivitis and atopic dermatitis, evaluating its therapeutic efficacy, adherence, and tolerability.

We examined three cases of allergic conjunctivitis associated with atopic dermatitis treated with once-daily 0.5% epinastine topical eyelid cream. Clinical outcomes were assessed using an allergic conjunctivitis severity score based on objective findings. Adherence and safety were also evaluated. All patients exhibited rapid symptom improvement, with significant reductions in ocular pruritus, conjunctival hyperemia, and eyelid dermatitis. By week four, allergic conjunctivitis severity scores had improved markedly in all cases (from baseline scores of 8-13 to 3-6). No adverse effects or treatment discontinuations were observed. Notably, all patients reported high adherence and ease of use, with reduced reliance on eye drops. This case series provides the clinical evidence supporting the efficacy and tolerability of 0.5% epinastine topical eyelid cream for allergic conjunctivitis, particularly in patients with coexisting atopic dermatitis. The once-daily application may enhance adherence and serve as a viable alternative or adjunct to conventional eye drops. Further large-scale studies are warranted to validate these findings.

## Introduction

Allergic conjunctivitis is a common ocular disorder that causes symptoms such as itching, redness, and excessive tearing, significantly impacting patients' quality of life (QOL). Conventional treatments primarily include anti-allergic and corticosteroid eye drops [1]. However, frequent dosing and adherence issues remain major challenges. For instance, a study of 62 patients undergoing treatment for allergic conjunctivitis reported that 21.0% of patients answered "mostly forget" (0-24%) when asked about adherence to the prescribed dosing regimen, while 61.3% responded "often forget" (25-74%), and 14.5% indicated "sometimes forget" (75-94%). Only 3.2% of patients reported "almost never forget" (95-100%) [2]. Ensuring treatment adherence is crucial for improving the therapeutic outcomes of chronic conditions. Most anti-allergic eye drops require administration two to four times per day, and reducing the frequency of application is likely to enhance adherence. Therefore, developing treatments with less frequent dosing may lead to improved compliance and better disease management.

In 2024, the world’s first once-daily applied cream formulation, 0.5% epinastine topical eyelid cream, was released as a treatment for allergic conjunctivitis [3-5]. This novel formulation, which requires only one application per day, represents a new type of anti-allergic medication. The long-lasting effect of the active ingredient is expected to improve patient adherence to treatment [4]. The introduction of this product provides a potential new treatment option for allergic conjunctivitis.

This report examines the clinical course, efficacy, and safety of 0.5% epinastine topical eyelid cream in the treatment of allergic conjunctivitis associated with atopic dermatitis in three cases. As a report from real-world clinical practice, this study discusses the potential utility of this treatment in managing allergic conjunctivitis in patients with atopic dermatitis.

## Case presentation

Methods

This study presents reports of three cases and was conducted in accordance with the ethical guidelines outlined in the Declaration of Helsinki (World Medical Association, 2013) and medical and healthcare ethics guidelines. Research related to the scoring of allergic conjunctivitis was approved by the Teikyo University Ethics Review Committee (#19-211) and registered as a clinical trial in the University Hospital Medical Information Network Clinical Trials Registry (UMIN-CTR) (registration numbers: UMIN000056319). All participants received a detailed explanation of the study's objectives and procedures, and written informed consent was obtained. The diagnosis of allergic conjunctivitis was made according to established guidelines [6,7].

The scoring of objective findings in allergic conjunctivitis was conducted according to the Japanese Allergic Conjunctival Disease Quality-of-Life Questionnaire (JACQLQ), with a total score ranging from 0 to 30 points [8-11].

First case

The patient was a five-year-old girl diagnosed with atopic dermatitis since infancy, with chronic dermatitis primarily affecting the face and neck. She had a history of seasonal allergic conjunctivitis, with symptom exacerbation during spring. For the past year, she had been using an anti-allergic eye drop (olopatadine) four times daily; however, due to frequent self-discontinuation, symptom control was inadequate.

During the cedar pollen season, she presented with bilateral eyelid swelling, severe itching, conjunctival hyperemia, and tearing. Initial ophthalmic examination revealed diffuse conjunctival hyperemia with mild papillary hypertrophy in both eyes. Tear fluid analysis showed elevated total IgE levels. Additionally, excoriation marks and scaling were observed on the eyelid skin. The allergic conjunctivitis severity score was 9 in the right eye and 8 in the left eye.

**Figure 1:**
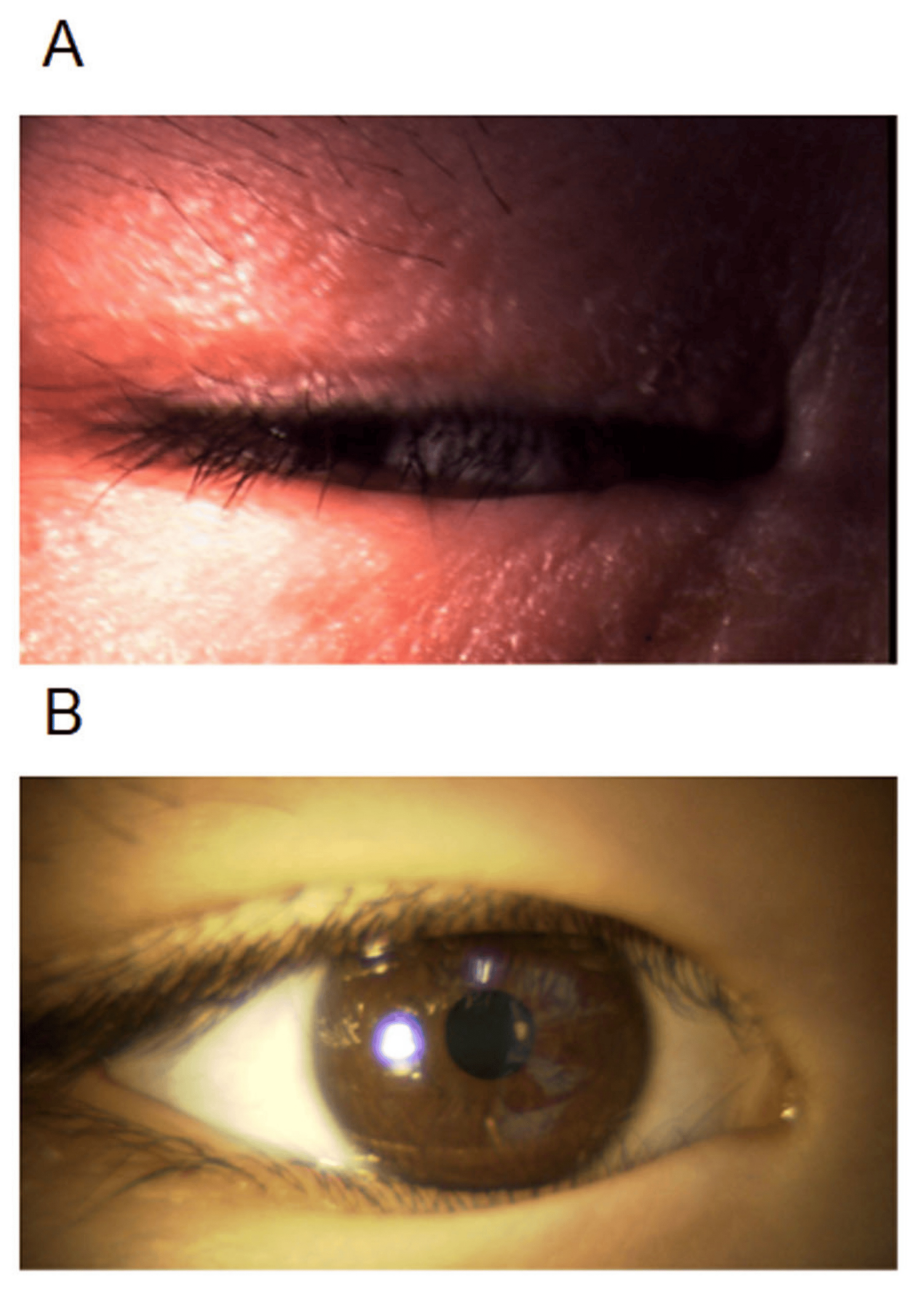
Anterior segment photographs of a five-year-old girl. Anterior segment photographs of the right eye show lid edema with minimal eyelid skin excoriation before treatment (A), and improvement at four weeks post-treatment with epinastine eyelid cream (B).

The patient was instructed to apply 0.5% epinastine topical eyelid cream once daily to the skin of both the upper and lower eyelids. Olopatadine eye drops were discontinued, and anti-allergic eye drops were not used during this period. After one week, pruritus was significantly alleviated, and secondary inflammation due to scratching was reduced. By four weeks, conjunctival hyperemia had nearly resolved, and excoriation marks and scaling on the eyelid skin had improved. Ultimately, the allergic conjunctivitis severity score improved to 3 in both eyes (Figures 1A, 1B). Epinastine eye cream was administered for one month until the end of the cedar pollen season, after which it was discontinued. Following the cessation of the cedar pollen season, no exacerbation of allergic conjunctivitis symptoms was observed.

Second case

The patient was an 82-year-old man with adult-onset atopic dermatitis, characterized by persistent eczema around the eyes and chronic allergic conjunctivitis. He had a known allergy to house dust and mites, suffering from persistent ocular pruritus and a foreign body sensation. He had previously been treated with antihistamine eye drops and topical corticosteroids; however, due to concerns about long-term steroid use, he had discontinued treatment on his own, leading to symptom worsening.

He presented with worsening ocular pruritus and foreign body sensation, particularly experiencing eyelid swelling upon waking. Initial ophthalmic examination revealed mild conjunctival hyperemia but prominent eyelid dermatitis, including eczematous lesions and scaling on the upper eyelids. Tear secretion was within normal limits, but total IgE levels in tear fluid were elevated. The allergic conjunctivitis severity score before treatment was 12 in the right eye and 11 in the left eye.

**Figure 2:**
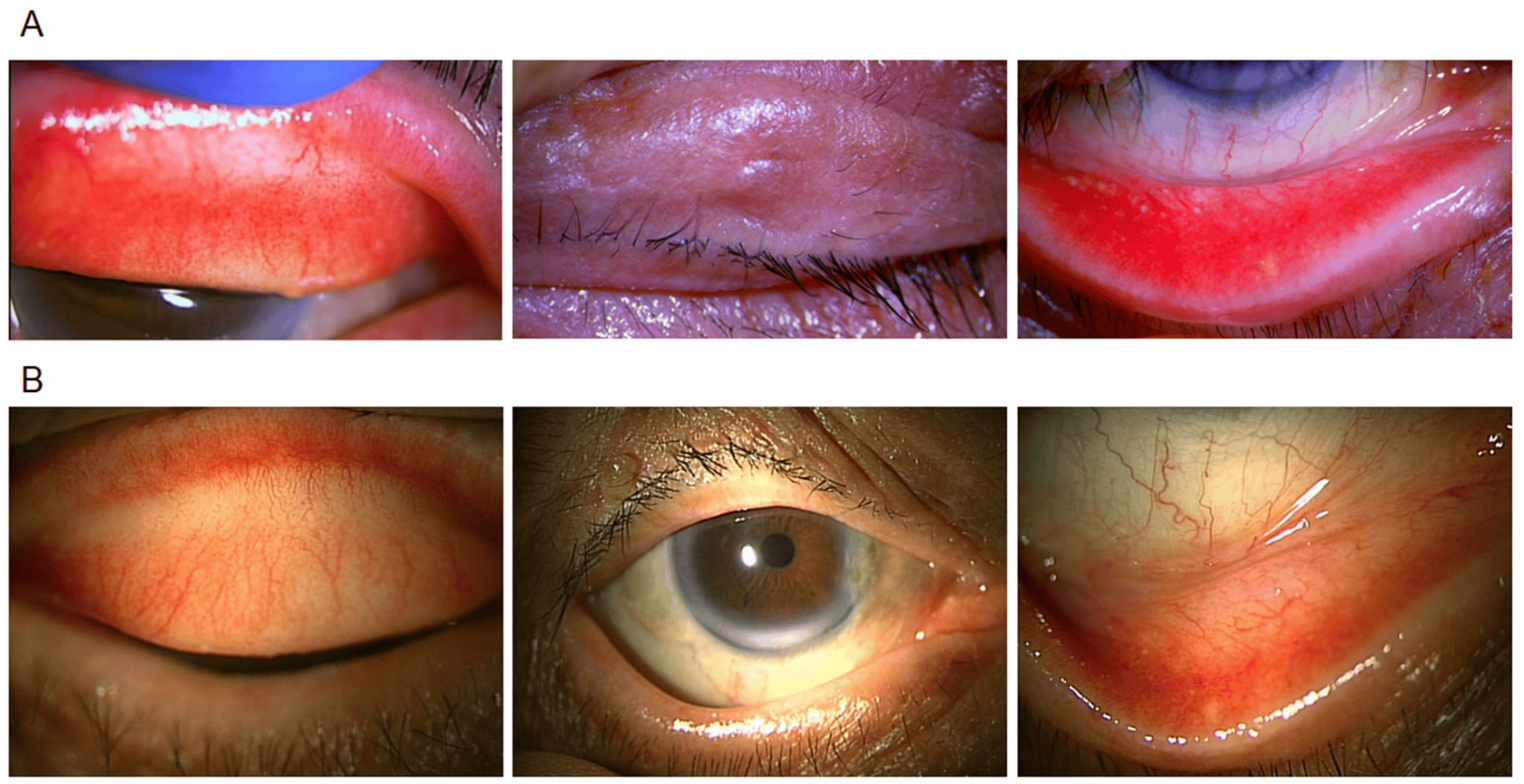
Anterior segment photographs of an 82-year-old male. Anterior segment photographs of the right eye before treatment (A), showing palpebral conjunctival congestion with few papillae, and at week four post-treatment with epinastine eyelid cream (B).

The patient was instructed to apply 0.5% epinastine topical eyelid cream once daily to the affected eyelid skin. After two weeks, eczematous changes of the eyelid improved, and pruritus decreased. By four weeks, the foreign body sensation had resolved, and restoration of skin barrier function was observed. The patient was able to manage symptoms without requiring topical corticosteroids, leading to a significant improvement in quality of life. At this point, the allergic conjunctivitis severity score had improved to 6 in the right eye and 5 in the left eye (Figures 2A, 2B).

Third case

This case involved a seven-year-old boy with allergic conjunctivitis triggered by cedar pollen in spring and ragweed pollen in autumn, along with a history of atopic dermatitis. He had been diagnosed with atopic dermatitis at the age of three, and skin eruptions on his hands and neck were still present. The patient did not require systemic medications, such as oral cetirizine or corticosteroids, for the management of atopic dermatitis. He developed allergic conjunctivitis at the age of four years, with symptom exacerbation predominantly in the autumn. Previous nonsteroidal eye drop treatments had provided insufficient relief, and he had discontinued their use over the past year.

He experienced worsening ocular pruritus and periorbital erythema, with symptoms intensifying at night. Examination revealed mild conjunctival hyperemia in both eyes, with a decreased noninvasive tear break-up time of 4 seconds bilaterally. The eyelid skin exhibited erythema and mild swelling, suggesting the influence of atopic dermatitis. A Dennie-Morgan fold was observed on the lower eyelid. Allergy testing showed an increased total IgE level in tear fluid, as well as elevated serum-specific IgE antibodies against ragweed pollen. The pre-treatment allergic conjunctivitis severity score was 13 in both eyes.

**Figure 3:**
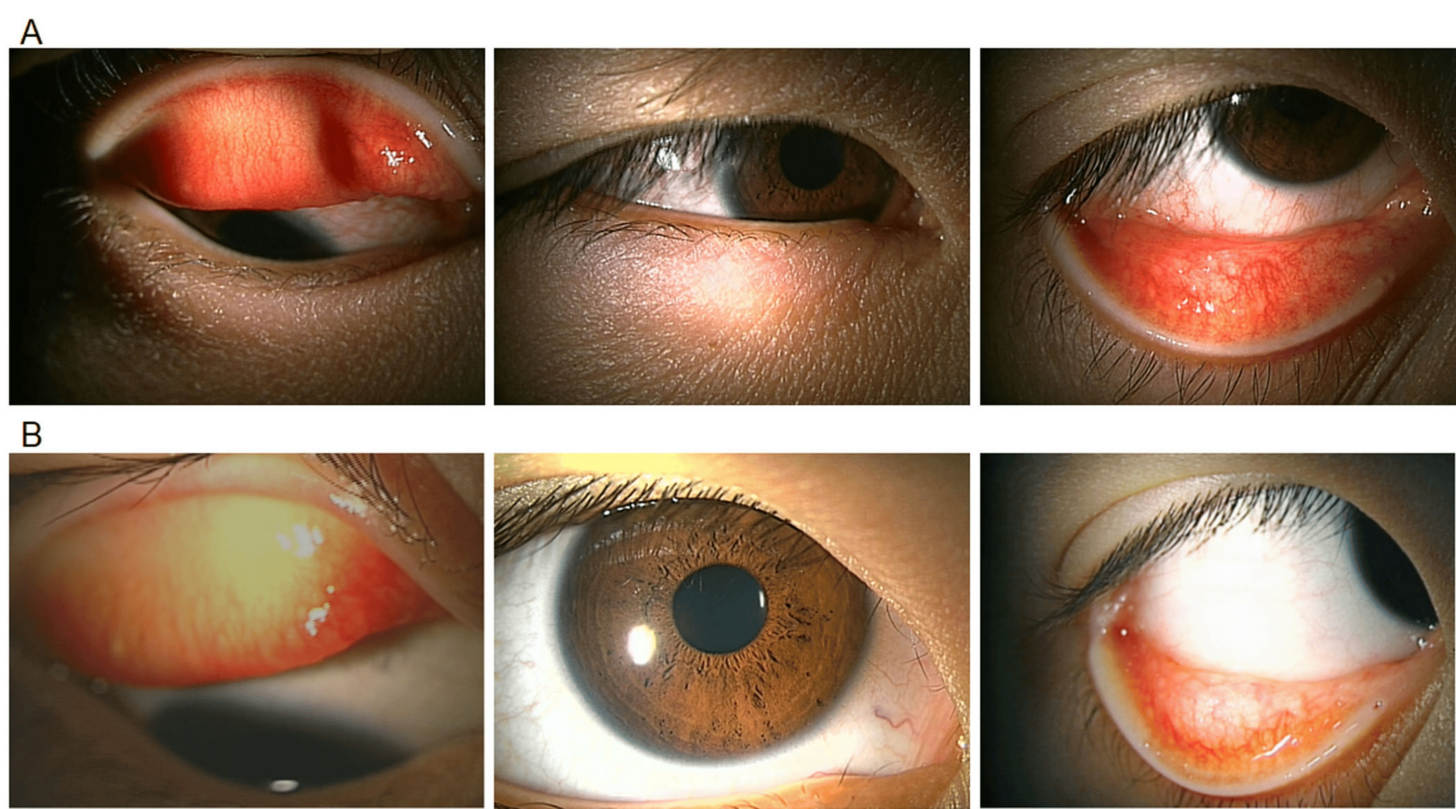
Anterior segment photographs of a seven-year-old boy. Anterior segment photographs of the right eye before treatment (A), showing palpebral conjunctival congestion, papillae, and lid edema with Dennie-Morgan fold, and at week four post-treatment with epinastine eyelid cream (B).

The patient was instructed to apply 0.5% epinastine topical eyelid cream once daily. After one week, nighttime ocular pruritus had markedly improved. By two weeks, eyelid erythema had resolved, and conjunctival hyperemia had diminished. The patient reported that the treatment was easy to use, reducing his dependence on eye drops. At week four post-treatment, the severity score of allergic conjunctivitis improved to 6 in both eyes (Figures 3A, 3B).

## Discussion

Summary of this study

This study presents three cases of allergic conjunctivitis with atopic dermatitis that were treated with once-daily application of 0.5% epinastine topical eyelid cream. The results suggest both efficacy and safety of this novel treatment. Compared to conventional ophthalmic solutions, this cream provides the unique advantage of simultaneously managing eyelid dermatitis and allergic conjunctivitis. Further long-term observations and large-scale clinical trials are necessary to establish its role within the therapeutic landscape.

Relationship between allergic conjunctivitis and atopic dermatitis

Allergic conjunctivitis and atopic dermatitis share common pathophysiological mechanisms, and their coexistence is not uncommon. In atopic dermatitis patients, impaired skin barrier function and chronic inflammation contribute to increased susceptibility to eyelid dermatitis, elevating the risk of secondary corneal and conjunctival complications, such as keratitis, corneal epithelial defects, and conjunctival hyperemia, due to frequent scratching. Studies have reported elevated levels of inflammatory cytokines, such as IL-4 and IL-13 and periostin, in the tear fluid of atopic dermatitis patients, which may exacerbate allergic responses on the ocular surface [12,13].

All three cases in this study involved patients with underlying atopic dermatitis who exhibited symptoms of both eyelid dermatitis and allergic conjunctivitis. In particular, mechanical damage to the eyelid skin from scratching may serve as a chronic conjunctival irritant, thereby prolonging allergic symptoms [14]. The use of 0.5% epinastine topical eyelid cream led to a notable improvement in both eyelid dermatitis, characterized by reduced erythema and pruritus, and allergic conjunctivitis symptoms including decreased ocular itching and redness. This dual therapeutic effect highlights its potential as a convenient treatment option for patients with overlapping allergic conditions.

Advantages of 0.5% epinastine topical eyelid cream

The 0.5% epinastine topical eyelid cream is the world’s first once-daily topical anti-allergic agent, offering several advantages over traditional ophthalmic or systemic treatments.

Conventional allergic conjunctivitis treatments typically require ophthalmic solutions to be administered two to four times daily, which poses a significant challenge for patient compliance. Eye drop administration requires proper technique, and self-administration can be particularly difficult for elderly individuals and children. Additionally, some patients discontinue use due to discomfort or irritation from preservatives in eye drops. In this report, all three patients had a history of difficulty adhering to ophthalmic therapy. The once-daily application of 0.5% epinastine topical eyelid cream resulted in high treatment adherence. Notably, two of the cases involved pediatric patients, suggesting the potential efficacy of this treatment for pediatric allergic conjunctivitis. The cream effectively alleviated nighttime symptoms and may serve as an alternative or adjunct to eye drops for patients with concurrent eyelid dermatitis.

In atopic dermatitis-associated allergic conjunctivitis, inflammation of the eyelid skin is known to exacerbate conjunctival symptoms [14]. Traditional treatments often involve a combination of topical corticosteroids for eyelid dermatitis and anti-allergic eye drops for allergic conjunctivitis. However, 0.5% epinastine topical eyelid cream offers the advantage of addressing both conditions with a single agent. In all three cases presented, eyelid inflammation improved following treatment, which coincided with a reduction in conjunctival symptoms. This suggests that suppressing skin inflammation may help mitigate secondary conjunctival irritation [14]. However, it is important to note that 0.5% epinastine topical eyelid cream is currently approved only for allergic conjunctivitis, not for eyelid dermatitis. Additionally, the favorable response observed in this study may be attributed to the mild severity of atopic dermatitis and the relatively low allergic conjunctivitis scores in these cases. In patients with extensive excoriation or severe epidermal disruption of the eyelid, ophthalmic ointments may be preferable.

Steroid eye drops are sometimes used to manage allergic conjunctivitis but are associated with risks such as elevated intraocular pressure and cataract formation with prolonged use. These risks are particularly concerning in pediatric patients, who are more susceptible to steroid-induced ocular hypertension. In contrast, 0.5% epinastine topical eyelid cream is a nonsteroidal anti-allergic agent, potentially reducing the risk of such adverse effects. In case 2, the patient had previously discontinued treatment due to concerns over long-term steroid use. However, following the introduction of 0.5% epinastine topical eyelid cream, symptom control was achieved without the need for corticosteroids. This suggests that this treatment may be a viable alternative for patients seeking to avoid steroid use.

Considerations when using 0.5% epinastine topical eyelid cream

While this treatment offers several advantages, certain precautions should be taken. The cream is designed for application to the eyelid skin and should not be applied directly to the conjunctiva or cornea. Accidental overapplication to the ocular surface may cause temporary irritation or blurred vision, necessitating proper patient education on its correct use.

Although no adverse effects were observed in the three cases presented, topical medications can sometimes cause skin irritation. Atopic dermatitis patients, in particular, have a compromised skin barrier and may exhibit increased sensitivity to topical formulations. It is advisable to monitor for potential skin reactions after initial use.

While 0.5% epinastine topical eyelid cream appears effective for mild-to-moderate allergic conjunctivitis, monotherapy may be insufficient for severe cases. In patients with giant papillary conjunctivitis or corneal complications, additional treatments such as anti-allergic eye drops, immunomodulatory agents, or steroid ophthalmic ointments may be necessary.

Limitations

This study describes a small observational study, which limits the generalizability of the findings regarding the efficacy and safety of 0.5% epinastine topical eyelid cream. Additionally, the lack of a control group prevents direct comparison with other treatment modalities. The short observation period also precludes assessment of long-term efficacy and safety. Future large-scale randomized controlled trials are required to further evaluate the clinical utility of this treatment.

## Conclusions

Topical eyelid cream containing 0.5 percent epinastine represents a promising new treatment option for the simultaneous management of allergic conjunctivitis and atopic dermatitis-related eyelid inflammation with once-daily application. This study suggests its high efficacy and good tolerability. However, appropriate guidance on application techniques and consideration of alternative treatments for severe cases are necessary.

As this report is based on a limited number of cases, further large-scale clinical trials are warranted to validate the efficacy and safety of 0.5% epinastine topical eyelid cream. Additionally, further research is needed to assess its long-term safety profile and its potential role in combination therapy with other anti-allergic treatments.
